# Strategies to improve treatment outcome in gastric cancer: A retrospective analysis of patients from two high-volume hospitals in Korea and China

**DOI:** 10.18632/oncotarget.9378

**Published:** 2016-05-15

**Authors:** Kun Yang, Yoon Young Choi, Wei-Han Zhang, Xin-Zu Chen, Mi Kyung Song, Jinae Lee, Bo Zhang, Zhi-Xin Chen, Hyoung-Il Kim, Jia-Ping Chen, Jae-Ho Cheong, Zong-Guang Zhou, Woo Jin Hyung, Jian-Kun Hu, Sung Hoon Noh

**Affiliations:** ^1^ Department of Gastrointestinal Surgery, West China Hospital, Sichuan University, Chengdu, China; ^2^ Laboratory of Gastric Cancer, State Key Laboratory of Biotherapy, West China Hospital, Sichuan University, Chengdu, China; ^3^ Department of Surgery, Severance Hospital, Yonsei University Health System, Yonsei University College of Medicine, Seoul, Republic of Korea; ^4^ Robot and Minimal Invasive Surgery Center, Severance Hospital, Yonsei University Health System, Seoul, Republic of Korea; ^5^ Biostatistics Collaboration Unit, Yonsei University College of Medicine, Seoul, Republic of Korea

**Keywords:** gastric cancer, gastrectomy, tumour characteristics, D2 lymphadenectomy, chemotherapy

## Abstract

China has high incidence of gastric cancer (GC). However, the treatment outcomes of China were unsatisfactory compared to those of Korea. We performed this study to compare tumour characteristics, treatment parameters, and survival outcomes of GC patients between Korea and China based on the databases of two high-volume hospitals, with the aim of identifying indicators of GC prognosis. Data of patients undergoing gastrectomy for GC from 2006 to 2010 were analysed retrospectively. Subgroup survival analyses, stratified by clinicopathologic factors and multivariable analyses, were performed. The interactive roles of chemotherapy and D2 lymphadenectomy for overall survival were also investigated. Among 1365 Chinese and 4981 Korean patients, the proportion of early cancer detection in Chinese patients was much lower relative to that of Korean patients. There were no significant differences between countries in terms of surgical morbidity and mortality. The overall 5-year survival rates were 54.3% and 81.4%; when stratified by clinicopathologic factors, the survival were generally statistically higher in Korean patients. Gender, age, T stage, N stage, extent of lymphadenectomy, radicality of surgery, resection type, and chemotherapy were independently associated with survival in patients without metastasis. Survival rates for stage II and III GC differed significantly between the two countries, but this difference was eliminated among patients who underwent D2 lymphadenectomy or received chemotherapy. These treatments were given to patients with advanced-stage diagnoses (approximately 20% and 80% of patients, respectively). Treatment type was selected as independent prognostic factors in stage I–III and D2/D2+, with chemotherapy resulting in the best prognosis. Many differences in GC tumour characteristics exist between two countries. Early cancer detection and standardized treatment in Korea contribute to superior survival rates. Promotion of an early screening program, training and dissemination of standard D2 lymphadenectomy, and appropriate applications of chemotherapy would improve survival outcomes.

## INTRODUCTION

The overall incidence of gastric cancer (GC) has been decreasing globally in recent years; however, it remains a disease with high incidence and mortality in East Asian countries [1]. China has a particularly high incidence of GC, accounting for nearly 45% of new GC cases and 40% of GC-related deaths worldwide [1, 2]. Compared with Korea, the long-term survival rates of GC patients, especially when diagnosed in advanced stages, are still not satisfactory in China, although they have improved in recent years. Despite the fact that GC is the most common cancer in Korea, its 5-year overall survival rates have been approximately 70% in recent years [3]. The reasons accounting for the survival differences between these two countries remain unclear and should be explored further. Furthermore, unsatisfactory treatment outcomes urge us to find effective strategies for treating GC in China. Considering the huge number of Chinese GC cases, improving treatment would significantly impact patients’ survival.

The survival rates after curative gastrectomy for GC is higher in East Asian countries than in Western countries [4–7]. Therefore, some studies have promoted the treatment of GC by comparing East Asian with Western countries. Because differences exist in intrinsic biological factors, diagnosis, and treatment of GC between Eastern and Western countries [4, 8, 9], some findings from Western countries have limitations when applied to China. However, Korean populations have similarities to Chinese populations in environment, genetic susceptibility to GC, and application of surgical procedures. Consequently, comparing Korea and China may be more meaningful than comparing Western and East Asian countries.

Therefore, we compared tumour characteristics, treatment parameters, and survival outcomes of GC patients between Korea and China based on the databases of two high-volume hospitals, with the aim of identifying factors that improve GC prognosis. To our knowledge, this is the first study to directly compare the characteristics and treatment outcomes of GC between Korea and China.

## RESULTS

### Clinicopathologic characteristics of patients

Clinicopathologic characteristics of patients are summarised in Table 1. The proportion of female patients in Korea was slightly higher than in Chinese patients. The percentage of patients with comorbidities was higher in Chinese patients. The locations of tumours were statistically different between these two countries. In Chinese patients, differentiation was poorer, tumours larger, depth of invasion greater, rate of lymph node involvement higher, and advanced stages more frequent relative to Korean patients. Distant metastasis was also more frequent in Chinese patients. The percentage of early GC (pT1, EGC) in Korean patients was much higher than that of Chinese patients. More Korean than Chinese patients were node-negative.

**Table 1:** General clinicopathological characteristics of the patients between different countries

	China (*N* = 1365)*	Korea (*N* = 4981)*	*P* value
**Gender**			0.001
Female	409 (30.0)	1737 (34.9)	
Male	956 (70.0)	3244 (65.1)	
**Age (yrs)**			0.118
< 60	768 (56.3)	2684 (53.9)	
≥ 60	597 (43.7)	2297 (46.1)	
**Comorbidity**	738 (54.1)	2427 (48.7)	< 0.001
**Tumor location**			< 0.001
Upper third	349 (25.6)	681 (13.7)	
Middle third	180 (13.2)	1365 (27.4)	
Lower third	806 (59.1)	2919 (58.6)	
Whole stomach	30 (2.2)	16 (0.3)	
**Differentiation**			< 0.001
G1	15 (1.1)	652 (13.1)	
G2	180 (13.2)	1415 (28.4)	
G3	1170 (85.7)	2914 (58.5)	
**Tumor size (cm)**			< 0.001
≤ 2	200 (14.7)	1665 (33.4)	
~5.0	645 (47.3)	2277 (45.7)	
~8.0	399 (29.2)	756 (15.2)	
> 8.0	121 (8.9)	283 (5.7)	
**Depth of infiltration (pT)**			< 0.001
pT1	231 (16.9)	2713 (54.5)	
pT2	172 (12.6)	555 (11.1)	
pT3	85 (6.2)	649 (13.0)	
pT4a	716 (52.5)	1031 (20.7)	
pT4b	161 (11.8)	33 (0.7)	
**Nodal status (pN)**			< 0.001
pN0	412 (30.2)	3124 (62.7)	
pN1	245 (18.0)	605 (12.2)	
pN2	222 (16.3)	514 (10.3)	
pN3a	302 (22.1)	457 (9.2)	
pN3b	184 (13.5)	281 (5.6)	
**Distal metastasis (M)**			< 0.001
M0	1221 (89.5)	4837 (97.1)	
M1	144 (10.6)	144 (2.9)	
**Stage**			< 0.001
I	281 (20.6)	2931 (58.8)	
II	264 (19.3)	797 (16.0)	
III	676 (49.5)	1109 (22.3)	
IV	144 (10.6)	144 (2.9)	

* Frequency (percentage).

### Treatment outcomes

Treatment outcomes are summarised in Table 2. A higher percentage of minimally invasive surgery, including laparoscopic surgery and robotic surgery, was conducted in Korea than China. Resection type differed significantly between countries, with distal gastrectomies performed more frequently in Korean patients and non-curative resections performed more frequently in Chinese patients. Lymphadenectomy approach also differed significantly between the countries. D1/D1^+^ lymphadenectomy was performed more often in Chinese than Korean patients; the reverse was true of D2/D2+ lymphadenectomy. The mean number of harvested lymph nodes was significantly lower in Chinese patients, not only overall, but also among patients who received a D2/D2+ lymphadenectomy. However, the overall postoperative morbidity rates and mortality were not significantly different between these two countries. Neo-adjuvant chemotherapy was rarely performed in both countries. Adjuvant treatment differed significantly between Chinese and Korean patients.

**Table 2:** Details of treatment and surgical short-term outcomes between different countries

	China (*N* = 1365)^†^	Korea (*N* = 4981)^†^	*P* value
**Surgical methods**			< 0.001
Open surgery	1176 (86.2)	3773 (75.8)	
Laparoscopic surgery	189 (13.9)	779 (15.6)	
Robotic surgery	0 (0)	429 (8.6)	
**Resection type**			< 0.001
Distal gastrectomy	793 (58.1)	3660 (73.5)	
Proximal gastrectomy	230 (16.9)	0 (0)	
Total gastrectomy	342 (25.1)	1321 (26.5)	
**Lymphadenectomy**			0.004
D1/D1+	689 (50.5)	2294 (46.1)	
D2/D2+*	676 (49.5)	2687 (53.9)	
**Radicality of surgery**			< 0.001
R0	1239 (90.8)	4780 (96.0)	
R1/R2	126 (9.2)	201 (4.0)	
**Reconstructions**			0.000
Billroth-1	192 (14.07%)	1853 (37.20%)	
Billroth-2	589 (43.15%)	1792 (35.98%)	
Roux-en-Y	354 (25.93%)	1336 (26.82%)	
Esophagogastric anastomosis	230 (16.85%)	0 (0%)	
**No. of total harvested lymph nodes**	27.22 ± 13.01	40.12 ± 16.03	< 0.001
**No. of total harvested lymph nodes in D2 subgroup**	30.00 ± 12.90	43.42 ± 16.43	< 0.001
**No. of lymph nodes with positive metastasis**	6.43 ± 8.39	3.07 ± 7.42	< 0.001
**Postoperative hospital stays (day)**	11.41 ± 7.75	9. 79 ± 9.36	0.000
**Estimated blood loss (mL) (4909 patients for analysis)**	172.00 ± 154.71	120.27 ± 192.66	0.000
**Operation time (min) (5806 patients for analysis)**	242.34 ± 54.38	169.16 ± 57.49	0.000
**Number of patients with postoperative morbidity**	200 (14.7)	708 (14.2)	0.682
**Clavien-Dindo classification of postoperative morbidity**			0.166
I	62 (4.5)	222 (4.5)	
II	75 (5.5)	240 (4.8)	
IIIa	42 (3.1)	156 (3.1)	
IIIb	10 (0.7)	70 (1.4)	
IVa	3 (0.2)	3 (0.1)	
IVb	2 (0.1)	5 (0.1)	
V	6 (0.4)	12 (0.2)	
**Mortality**	6 (0.4)	12 (0.2)	0.221
**Chemotherapy**			< 0.001
No chemotherapy	764 (56.0)	3411 (68.5)	
Neo-adjuvant chemotherapy	12 (0.9)	87 (1.7)	
Adjuvant chemotherapy	575 (42.1)	1408 (28.3)	
Unclear	14 (1.0)	75 (1.5)	

* D2 included No.14v lymph nodes dissection.

† Frequency (percentage).

### Prognosis of GC

**Figure 1:**
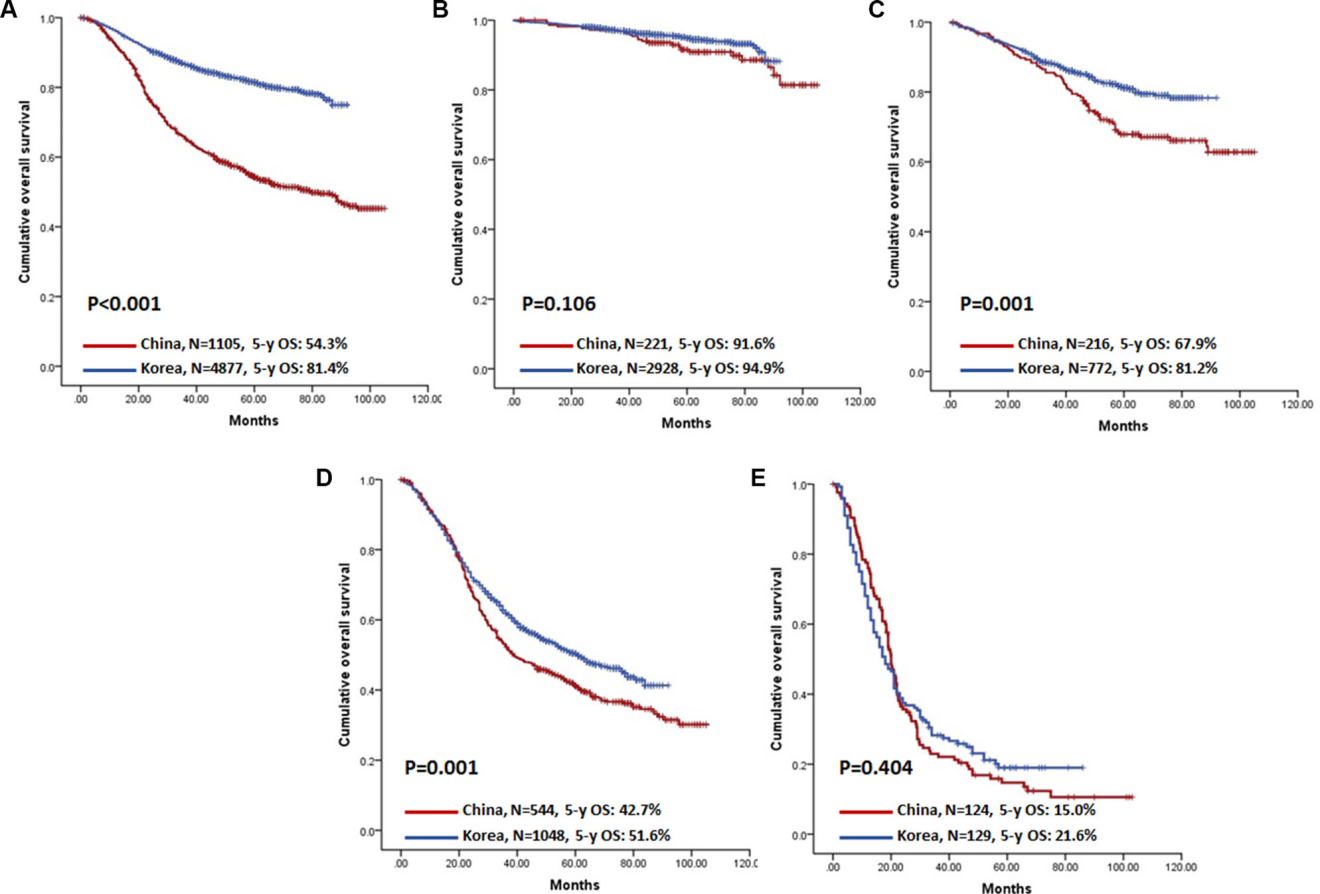
Kaplan-Meier survival analysis of patients between China and Korea (**A**) Overall patients. (**B**–**E**) Patients stratified by stage. (B) Stage I patients; (C) Stage II patients; (D) Stage III patients; (E) Stage IV patients.

The 5-year OS for Chinese and Korean patients was 54.3% and 81.4%, respectively (*P* < 0.001, Figure 1A). When stratified by clinicopathologic characteristics and treatment parameters, the prognoses of Korean patients were substantially better than those of Chinese patients in most subgroup analyses; in advanced GC subgroups, such as those receiving R1/R2 resection, whole stomach lesion, T4b, N3b, or with distant metastasis, the OS of Chinese patients was not significantly different from that of Korean patients (Table 3). Prognosis was also compared between countries according to stage, and OS was significantly lower among Chinese stage II and III patients (Table 3 and Figure 1B–1E).

**Table 3:** Survival analyses stratified by clinicopathologic characteristics and treatment parameters in different countries

	China (*N* = 1105)		Korea (*N* = 4877)		*P* value^†^
	*N*	5-y OSR (%)	*N*	5-y OSR (%)	
**Gender**					
Female	324	56.3	1700	83.3	< 0.001
Male	781	53.4	3177	80.4	< 0.001
**Age (yrs)**					
< 60	618	55.4	2643	84.5	< 0.001
≥ 60	487	52.9	2234	77.6	< 0.001
**Longitudinal Tumor location**					
Upper third	280	43.7	670	76.9	< 0.001
Middle third	140	54.4	1334	82.4	< 0.001
Lower third	661	59.9	2861	82.1	< 0.001
Whole stomach	24	23.4	12	43.7	0.155
**Differentiation**					
G1	11	90.0	645	92.0	0.814
G2	148	62.7	1387	81.0	< 0.001
G3	946	52.6	2845	79.1	< 0.001
**Tumor size (cm)**					
≤ 2	157	83.8	1660	94.7	< 0.001
~ 5.0	523	59.2	2232	83.0	< 0.001
~ 8.0	327	40.0	719	62.6	< 0.001
> 8.0	98	28.1	266	40.3	0.026
**Depth of infiltration (pT)**					
pT1	180	91.4	2710	95.0	0.056
pT2	150	71.4	547	85.0	0.001
pT3	73	54.3	621	75.3	< 0.001
pT4a	573	44.4	970	47.7	0.352
pT4b	129	26.1	29	23.6	0.278
**Nodal status (pN)**					
pN0	320	80.9	3111	92.8	< 0.001
pN1	200	61.8	583	84.1	< 0.001
pN2	184	60.1	493	68.9	0.020
pN3a	245	32.8	432	45.1	0.001
pN3b	156	16.8	258	22.9	0.249
**Distal metastasis (M)**					
M0	981	59.2	4748	83.0	< 0.001
M1	124	15.0	129	21.6	0.404
**Stage**					
I	221	91.6	2928	94.9	0.106
II	216	67.9	772	81.2	0.001
III	544	42.7	1048	51.6	0.001
IV	124	15.0	129	21.6	0.404
**Surgical methods**					
Open surgery	943	52.9	3675	78.1	< 0.001
Minimal invasive surgery	162	62.2	1202	92.0	< 0.001
**Resection type**					
Subtotal gastrectomy	827	59.1	3606	86.1	<0.001
Total gastrectomy	278	40.0	1271	68.0	<0.001
**Lymphadenectomy**					
D1/D1+	578	49.4	2278	91.3	< 0.001
D2/D2+*	527	59.6	2599	72.3	< 0.001
**Radicality of surgery**					
R0	996	58.6	4695	83.6	< 0.001
R1/R2	109	14.4	182	25.7	0.065
**Chemotherapy**					
No	593	50.0	3396	91.5	< 0.001
Yes	512	59.2	1481	57.9	0.347

Abbreviations: OSR, overall survival rate.

* D2 included No.14v lymph nodes dissection.

† *p*-value for log-rank test.

### Independent prognostic factors

**Figure 2:**
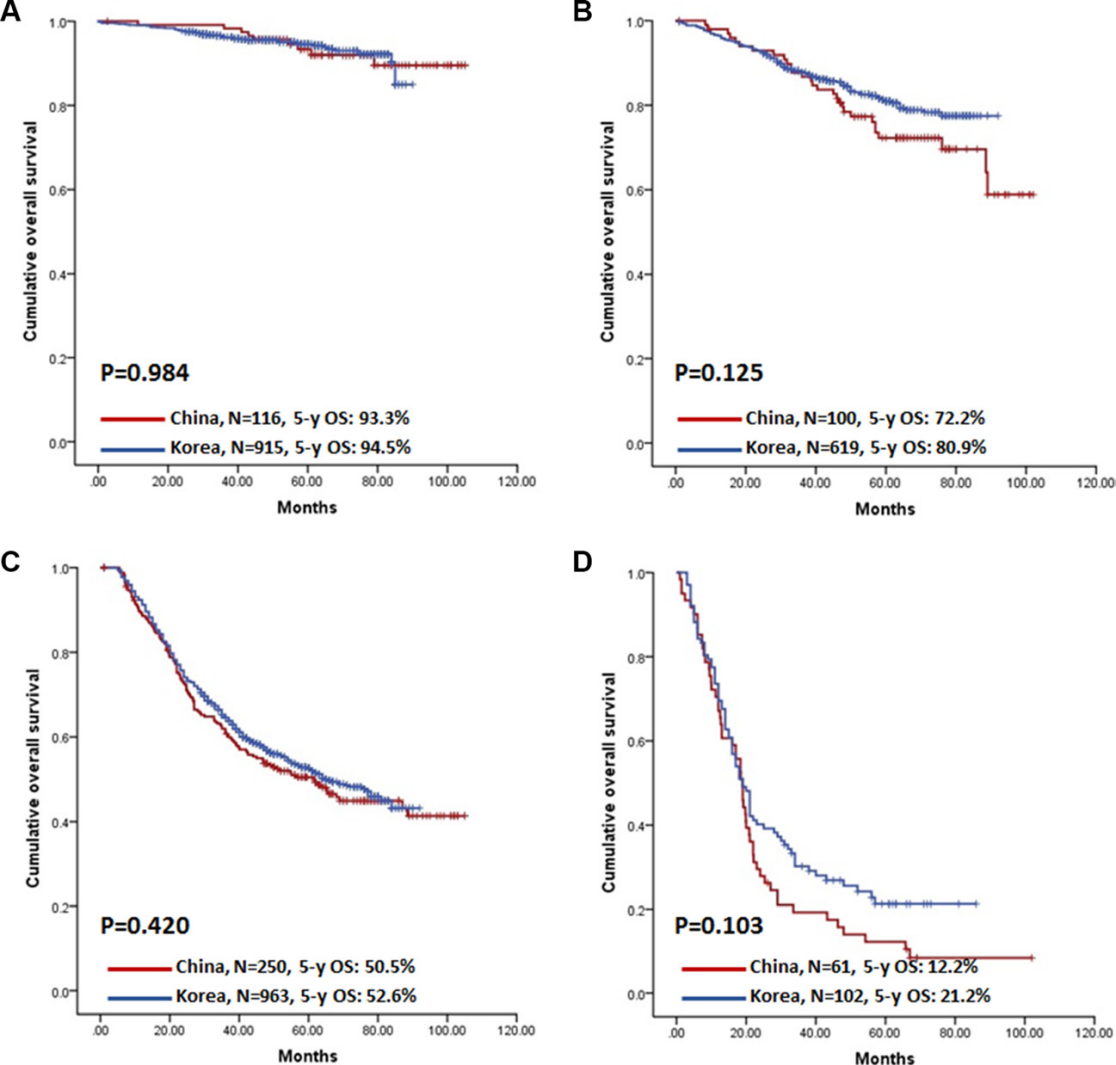
Kaplan-Meier survival analysis of patients with D2 or D2+ lymphadenectomy stratified by stage between China and Korea (**A**) Stage I patients; (**B**) Stage II patients; (**C**) Stage III patients; (**D**) Stage IV patients.

**Figure 3:**
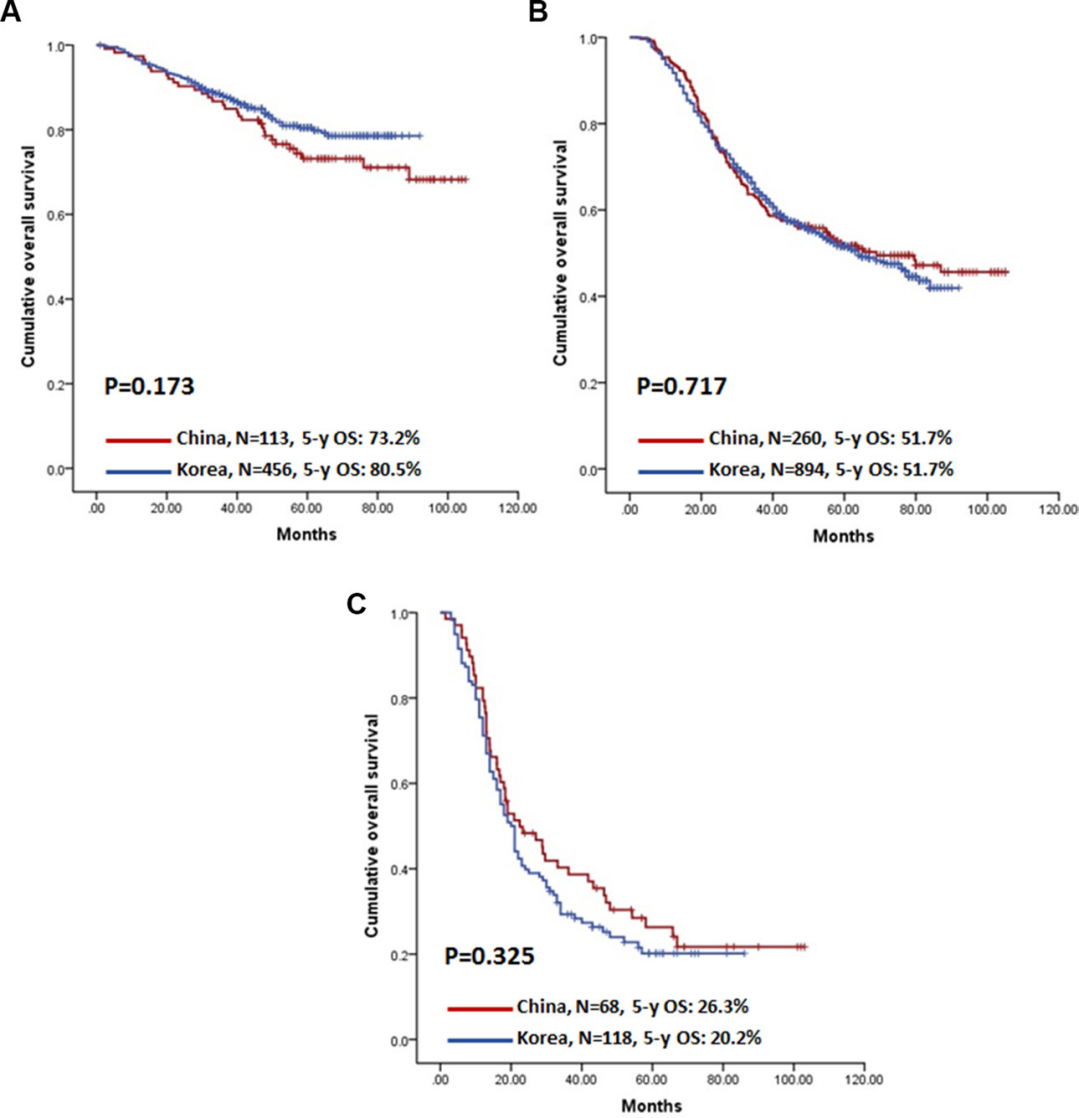
Kaplan-Meier survival analysis of patients with chemotherapy stratified by stage between China and Korea (**A**) Stage II patients; (**B**) Stage III patients; (**C**) Stage IV patients.

Because the prognoses differed in Chinese and Korean patients, even for patients in the same TNM stages (especially for stage II and III), further analyses focused on identifying independent prognostic factors in overall. The analysis classified patients into two groups: those without distant metastasis (M0) and those with metastasis (M1). In M0 patients, gender, age, depth of infiltration, nodal status, extent of lymphadenectomy, radicality of surgery, resection type, and chemotherapy were independently associated with prognosis (Table 4). In M1 patients, tumour size and chemotherapy were independent prognostic factors. Among these independent prognostic factors, lymphadenectomy and chemotherapy were the only clinically-correctable factors; thus, we extended our analysis to compare prognoses in patients who underwent D2/D2+ lymphadenectomy (Figure 2A–2D) or received adjuvant chemotherapy (Figure 3A–3C). Intriguingly, the prognostic differences between Chinese and Korean patients were attenuated when D2/D2+ lymphadenectomy and chemotherapy were analysed in isolation. Furthermore, country was not selected by multivariable analyses as an independent prognostic factor in either the M0 or M1 group (Table 4).

**Table 4:** The prognostic factors on the univariable and multivariable cox-proportional hazard regression analyses

	M0 patients (*N* = 5729)				M1 patients (*N* = 253)			
	Univariable HR (95% CI)	*P* value	Multivariable HR* (95% CI)	*P* value	Univariable HR (95% CI)	*P* value	Multivariable HR* (95% CI)	*P* value
**Country**		< 0.001				0.409		
China	1				1			
Korea	0.37 [0.33–0.42]				0.89 [0.68–1.17]			
**Gender**		< 0.001		0.011		0.125		
Male	1		1		1			
Female	0.75 [0.66–0.85]		0.85 [0.74–0.96]		1.25 [0.94–1.66]			
**Age (yrs)**		< 0.001		< 0.001		0.118		
< 60	1		1		1			
= 60	1.48 [1.32–1.66]		1.50 [1.33–1.68]		0.80 [0.60–1.06]			
**Tumor location**		< 0.001				0.676		
Upper third	1				1			
Middle third	0.57 [0.48–0.68]	< 0.001			0.95 [0.64–1.42]	0.794		
Lower third	0.66 [0.57–0.76]	< 0.001			0.83 [0.59–1.18]	0.309		
Whole stomach	3.29 [1.92–5.63]	< 0.001			1.07 [0.57–2.02]	0.839		
**Differentiation**		< 0.001				0.069		
G1	1				1			
G2	2.78 [2.04–3.79]	< 0.001			3.58 [0.48–26.58]	0.212		
G3	3.66 [2.73–4.91]	< 0.001			5.20 [0.73–37.16]	0.101		
**Tumor size (cm)**		< 0.001				< 0.001		< 0.001
= 2	1				1		1	
~5.0	3.30 [2.69–4.06]	< 0.001			1.08 [0.43–2.72]	0.869	1.12 [0.45–2.82]	0.810
~8.0	7.70 [6.23–9.52]	< 0.001			1.93 [0.78–4.77]	0.152	1.90 [0.77–4.70]	0.163
>8.0	12.28 [9.65–15.63]	< 0.001			2.65 [1.06–6.62]	0.036	2.64 [1.06–6.58]	0.038
**Depth of infiltration (pT)**		< 0.001		< 0.001		0.188		
pT1	1		1		1			
pT2	3.56 [2.80–4.52]	< 0.001	2.89 [2.23–3.74]	< 0.001	8.37 [0.87–80.81]	0.066		
pT3	5.51 [4.42–6.87]	< 0.001	3.60 [2.78–4.67]	< 0.001	3.08 [0.40–23.70]	0.280		
pT4a	12.04 [10.07–14.38]	< 0.001	6.07 [4.80–7.66]	< 0.001	5.08 [0.71–36.36]	0.106		
pT4b	23.61 [17.83–31.27]	< 0.001	7.31 [5.29–10.10]	< 0.001	4.74 [0.65–34.44]	0.124		
**Nodal status (pN)**		< 0.001		< 0.001		0.063		
pN0	1		1		1			
pN1	2.65 [2.17–3.24]	< 0.001	1.49 [1.20–1.85]	< 0.001	1.12 [0.53–2.36]	0.759		
pN2	4.62 [3.86–5.54]	< 0.001	2.10 [1.70–2.59]	< 0.001	0.98 [0.46–2.09]	0.958		
pN3a	9.94 [8.46–11.68]	< 0.001	3.90 [3.18–4.77]	< 0.001	1.24 [0.65–2.37]	0.506		
pN3b	17.34 [14.49–20.75]	< 0.001	6.32 [5.05–7.90]	< 0.001	1.71 [0.92–3.19]	0.093		
**Lymphadenectomy**		< 0.001		0.015		0.288		
D1/D1+	1		1		1			
D2/D2+	1.91 [1.70–2.15]		0.85 [0.74–0.97]		1.17 [0.88–1.56]			
**Radicality of surgery**		< 0.001		< 0.001		0.578		
R0	1		1		1			
R1/R2	5.00 [3.84–6.50]		1.84 [1.40–2.41]		1.10 [0.78–1.56]			
**Resection type**		< 0.001		0.011		0.004		
Subtotal	1		1		1			
Total	2.03 [1.81–2.29]		1.17 [1.04–1.32]		1.50 [1.14–1.98]			
**Chemotherapy**		< 0.001		0.001		0.002		0.008
No	1		1		1		1	
Yes	3.11 [2.78–3.49]		0.79 [0.69–0.91]		0.62 [0.46–0.84]		0.66 [0.49–0.90]	

Abbreviations: HR, hazard ratio.

* forward selection with likelihood ratio.

### Effects of lymphadenectomy and chemotherapy by GC stage

To scrutinize the effects of lymphadenectomy and chemotherapy across all stages, 5-year OS was compared at each stage (Table 5). The benefit of chemotherapy was observed mainly in stage III and IV, and the prognosis of the chemotherapy group was significantly better than the no-chemotherapy group in stage II when D1/D1+ lymphadenectomy was performed. When the benefit of D2/D2+ vs. D1/D1+ lymphadenectomy was compared, the prognosis of the D2/D2+ group was better in stage III, but not stages I, II, or IV. Intriguingly, D2/D2+ lymphadenectomy was positively related to prognosis in stage II when the patients did not receive chemotherapy.

**Table 5:** Survival of patients stratified by stages according to lymphadenectomy and chemotherapy in overall population (*N* = 5982)

	Ngroup 1	5-y OSgroup 1	Ngroup 2	5-y OSgroup 2	*P* value^†^	Unadjusted HR (95% CI)	*P* value	Adjusted HR (95% CI)*	*P* value	Adjusted HR (95% CI)* for patients with R0 resection	*P* value
**Without chemotherapy (Group 1) VS with chemotherapy (Group 2)**											
I	3065	94.7%	84	90.4%	0.162	1.60 [0.82–3.11]	0.165	1.22 [0.54–2.71]	0.635	1.34 [0.60–2.96]	0.474
II	419	76.4%	569	78.6%	0.404	0.89 [0.68–1.17]	0.405	0.84 [0.62–1.14]	0.255	0.83 [0.61–1.12]	0.226
III	438	40.2%	1154	51.8%	< 0.001	0.70 [0.60–0.81]	< 0.001	0.69 [0.59–0.81]	< 0.001	0.68 [0.57–0.80]	< 0.001
IV	67	7.3%	186	22.4%	0.001	0.62 [0.45–0.84]	0.002	0.60 [0.42–0.86]	0.006	0.33 [0.13–0.87]	0.025
**Group 1 VS Group 2 in D2/D2+ lymphadenectomy group**											
I	993	94.3%	38	92.0%	0.983	1.01 [0.31–3.28]	0.983	1.21 [0.29–5.00]	0.794	1.21 [0.29–5.00]	0.794
II	304	80.5%	415	78.5%	0.531	1.12 [0.79–1.57]	0.532	1.05 [0.71–1.55]	0.814	1.06 [0.72–1.56]	0.785
III	266	46.4%	947	53.9%	0.007	0.78 [0.64–0.94]	0.008	0.70 [0.57–0.86]	0.001	0.69 [0.56–0.85]	< 0.001
IV	33	3.0%	130	21.5%	0.003	0.55 [0.37–0.83]	0.004	0.53 [0.31–0.91]	0.021	–^#^	–^#^
**Group 1 VS Group 2 in D1/D1+ lymphadenectomy group**											
I	2072	95.0%	46	89.0%	0.060	2.14 [0.95–4.79]	0.065	1.40 [0.51–3.87]	0.515	1.42 [0.52–3.93]	0.497
II	115	65.6%	154	79.0%	0.010	0.53 [0.33–0.87]	0.011	0.51 [0.30–0.89]	0.018	0.51 [0.30–0.89]	0.018
III	172	30.6%	207	42.7%	0.001	0.67 [0.52–0.86]	0.002	0.60 [0.45–0.80]	< 0.001	0.60 [0.45–0.82]	0.001
IV	34	11.4%	56	24.0%	0.044	0.62 [0.38–0.99]	0.047	0.43 [0.24–0.78]	0.005	–^#^	–^#^
**D1/D1+ (Group 1) VS D2/D2+ lymphadenectomy (Group 2)**											
I	2118	94.8%	1031	94.2%	0.323	1.18 [0.85–1.62]	0.324	0.94 [0.66–1.34]	0.720	0.98 [0.68–1.39]	0.888
II	269	73.3%	719	79.1%	0.186	0.80 [0.60–1.08]	0.146	0.78 [0.56–1.10]	0.160	0.76 [0.54–1.07]	0.118
III	379	37.2%	1213	52.2%	< 0.001	0.70 [0.60–0.81]	< 0.001	0.79 [0.66–0.95]	0.014	0.81 [0.67–0.99]	0.040
IV	90	19.3%	163	17.8%	0.283	1.17 [0.88–1.56]	0.288	1.39 [0.98–1.97]	0.066	2.64 [0.96–7.26]	0.060
**Group 1 VS Group 2 in with chemotherapy group**											
I	46	89.0%	38	92.0%	0.231	0.44 [0.11–1.74]	0.243	0.16 [0.015–1.68]	0.127	0.16 [0.015–1.68]	0.127
II	154	79.0%	415	78.5%	0.535	1.14 [0.75–1.75]	0.535	1.07 [0.65–1.77]	0.787	1.05 [0.64–1.74]	0.837
III	207	42.7%	947	53.9%	0.022	0.79 [0.64–0.97]	0.023	0.73 [0.57–0.93]	0.011	0.74 [0.57–0.97]	0.025
IV	56	24.0%	130	21.5%	0.291	1.22 [0.84–1.75]	0.296	1.07 [0.68–1.69]	0.763	–^#^	–^#^
**Group 1 VS Group 2 in without chemotherapy group**											
I	2072	95.0%	993	94.3%	0.196	1.24 [0.89–1.72]	0.197	0.95 [0.66–1.37]	0.786	0.98 [0.68–1.41]	0.900
II	115	65.6%	304	80.5%	0.004	0.54 [0.35–0.81]	0.003	0.56 [0.34–0.93]	0.023	0.56 [0.34–0.93]	0.023
III	172	30.6%	266	46.4%	0.002	0.69 [0.54–0.88]	0.002	0.67 [0.48–0.93]	0.016	0.69 [0.50–0.95]	0.025
IV	34	11.4%	33	3.0%	0.068	1.59 [0.96–2.64]	0.074	1.79 [0.96–3.34]	0.069	–^#^	–^#^

Abbreviations: N, number; OS, overall survival; HR, hazard ratio.

Note: The values of group 1 were regarded as references for the HR analyses.

† p-value for log-rank test.

* Adjust for country, gender, age, surgical methods, tumor location, differentiation, tumor size, T stage, N stage, resection type, lymphadenectomy, radicality of surgery, chemotherapy.

# The valid sample size is too small to calculate the adjust HR.

### Different treatment in different countries and the possible benefit of D2/D2+ lymphadenectomy and chemotherapy in China

**Figure 4:**
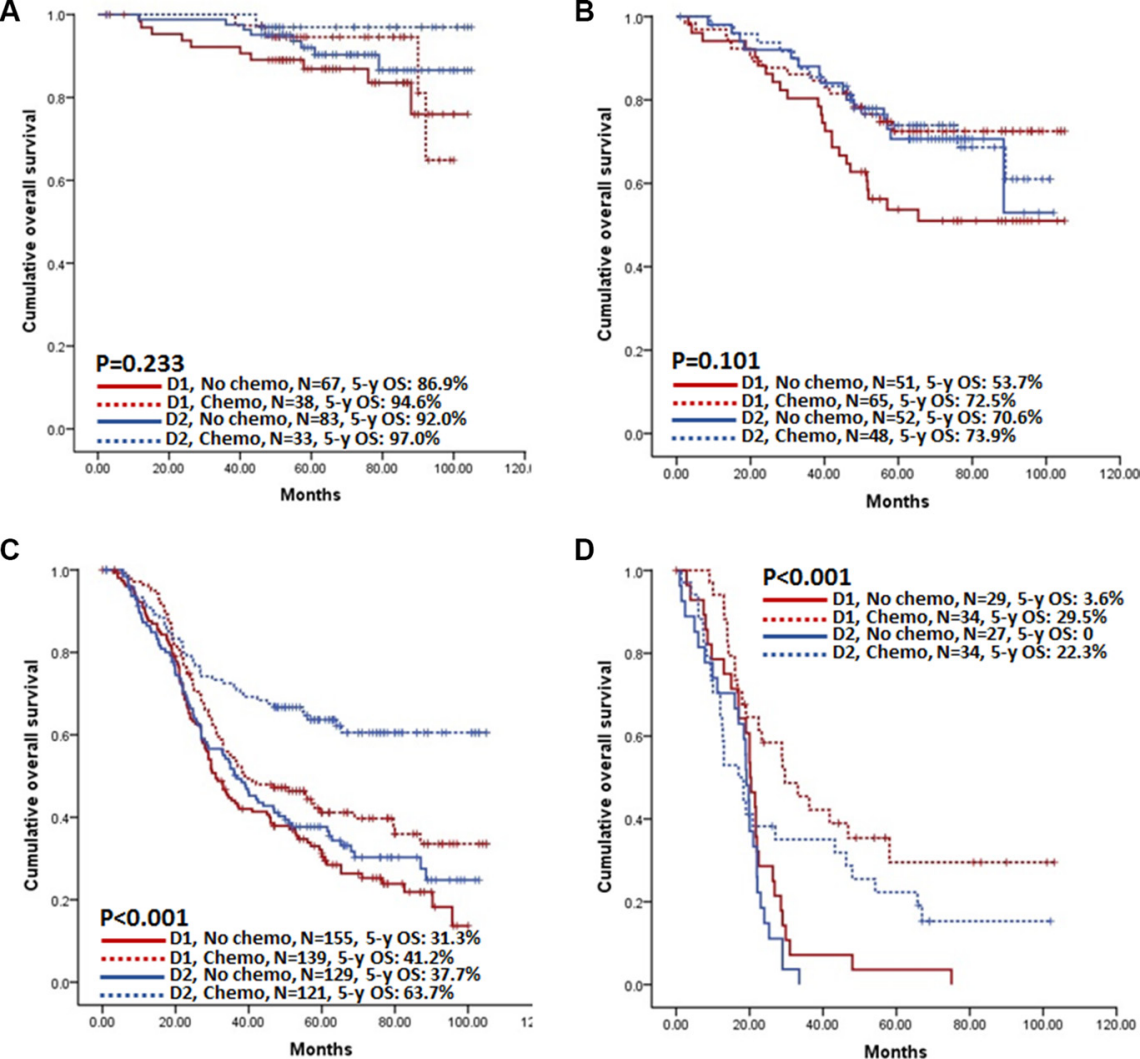
Kaplan-Meier survival analysis of Chinese patients according to the treatment type in each stage (**A**) Stage I patients; (**B**) Stage II patients; (**C**) Stage III patients; (**D**) Stage IV patients.

We assessed the proportions of patients received D2/D2+ lymphadenectomy and/or chemotherapy, stratifying these independent and clinically-correctable prognostic factors by stage in both countries (Table 6). Approximately 50% of Chinese patients with advanced disease (stage III and IV) received D2/D2+ lymphadenectomy or chemotherapy, while over 80% of patients in Korea. Only around 20% of Chinese patients were treated by both D2/D2+ lymphadenectomy and chemotherapy; but over 60% of patients in Korea. When comparing the benefit of the combination of D2/D2+ lymphadenectomy and chemotherapy according to each stage in Chinese patients, D1/D1+ lymphadenectomy without chemotherapy in stage II and III had the poorest prognosis, whereas the benefit from D2/D2+ lymphadenectomy with chemotherapy was prominent in stage III (Figure 4A–4D). In multivariable analyses, treatment type was selected as an independent prognostic factor in M0 patients, where D2/D2+ lymphadenectomy with chemotherapy showed the best prognosis (Table 7). Treatment type was also an independent prognostic factor in M1 patients. However, only chemotherapy was positively correlated to better prognosis; D2/D2+ lymphadenectomy was not.

**Table 6:** Multivariable cox-proportional hazard regression analysis in Chinese population (*N* = 1105)

	China				Korea			
	Total number of patients	Number of Patients with D2/D2+ (%)	Number of Patients with chemotherapy (%)	Number of Patients with D2/D2+ and chemotherapy (%)	Total number of patients	Number of Patients with D2/D2+ (%)	Number of Patients with chemotherapy (%)	Number of Patients with D2/D2+ and chemotherapy (%)
**Stage I**	281	149 (53.0)	82 (29.2)	39 (13.9)	2931	917 (31.3)	13 (0.4)	5 (0.2)
**Stage II**	264	130 (49.2)	131 (49.6)	58 (22.0)	797	640 (80.3)	456 (57.2)	367 (46.0)
**Stage III**	676	325 (48.1)	298 (44.1)	141 (20.9)	1109	1021 (92.1)	904 (81.5)	836 (75.4)
**Stage IV**	144	72 (50.0)	76 (52.8)	36 (25.0)	144	109 (75.7)	122 (84.7)	96 (66.7)

**Table 7:** Multivariable cox-proportional hazard regression analysis in Chinese population (*N* = 1105)

M0 patients (*N* = 981)			M1 patients (*N* = 124)		
	Multivariable HR*	*P* value		Multivariable HR^†^	*P* value
**Treatment**		< 0.001	**Treatment**		0.001
D1/D1+ without chemotherapy	1		D1/D1+ without chemotherapy	1	
D1/D1+ with chemotherapy	0.63 [0.49–0.81]	< 0.001	D1/D1+ with chemotherapy	0.45 [0.25–0.79]	0.006
D2/D2+ without chemotherapy	0.86 [0.67–1.11]	0.244	D2/D2+ without chemotherapy	1.53 [0.88–2.63]	0.129
D2/D2+ with chemotherapy	0.44 [0.32–0.59]	< 0.001	D2/D2+ with chemotherapy	0.91 [0.53–1.57]	0.732
**Radicality of surgery**		0.001	**Tumor size (cm)**		0.005
R0	1		≤ 2	1	
R1/R2	1.97 [1.30–2.99]		~5.0	3.43 [0.46–25.78]	0.232
**Depth of infiltration (pT)**		< 0.001	~8.0	5.81 [0.78–43.40]	0.087
pT1	1		> 8.0	7.73 [1.02–58.64]	0.048
pT2	2.28 [1.32–3.95]	0.003			
pT3	3.31 [1.84–5.94]	< 0.001			
pT4a	3.80 [2.34–6.18]	< 0.001			
pT4b	4.18 [2.39–7.32]	< 0.001			
**Nodal status (pN)**		< 0.001			
pN0	1				
pN1	1.82 [1.28–2.59]	0.001			
pN2	1.80 [1.26–2.57]	0.001			
pN3a	3.92 [2.83–5.43]	< 0.001			
pN3b	5.25 [3.64–7.57]	< 0.001			

Abbreviations: HR, hazard ratio.

* forward selection (likelihood ratio) with treatment type, gender, age, surgical methods, tumor location, differentiation, tumor size, T stage, N stage, resection type, radicality of surgery.

† forward selection (likelihood ratio) with treatment type, gender, age, surgical methods, tumor location, differentiation, tumor size, T stage, N stage, M stage, resection type, radicality of surgery.

## DISCUSSION

In our analyses, the 5-year OS rate of Korean GC patients was substantially higher than that of Chinese patients. We performed multivariable analyses to identify the prognostic factors that most influenced survival. After adjustment, country was not a significant independent prognostic factor. Of the factors identified as independent prognostic factors, some are beyond medical control (including gender, age, depth of infiltration, lymph node metastasis, and resection type), whereas other factors are capable of influence by doctors (including lymphadenectomy, radicality of surgery, and chemotherapy). To our knowledge, this is the first study, not only emphasizing the different cancer characteristics between the two countries, but also focusing on the roles of doctor-correctable factors. This is also the first study to directly propose the solutions to improve the treatment of GC in China.

The proportion of early cancers was much lower and the advanced stages were more frequent in Chinese patients. Conversely, nearly 60% of Korean patients were diagnosed within early stages of GC. Therefore, it is intuitive that the higher proportion of advanced-stage patients contributed to the poorer survival of Chinese patients. Thanks to the national, population-based GC screening program established in Korea in 1999 [10], the percentage of GC diagnosed in early stage has gradually increased from 15% during 1974–1992 to 58% in 2009 [11]. In recent years, despite a slight upward trend of early GC detection in China due to the availability of upper gastrointestinal endoscopy and a shift towards health- consciousness in China [7, 12, 13], the proportion of early GC diagnoses is still low, and advanced GC remains the leading health burden and cause of cancer-related mortality. Fortunately, annual reports indicate that the incidence and mortality of GC has decreased and the mortality-to-prevalence ratio of GC has tended to decline in recent years [11]. These trends have resulted from the China Cancer Prevention and Control Program, a governmental platform dedicated to prevention, screening, and surveillance for cancers (including GC), although there is not a formal, specialized, nationwide cancer-screening program [11]. Establishment of a formal and specialized nationwide screening program for GC will further promote early detection, which will also mitigate the extent of medically-uncontrollable factors that impact survival. In addition, early detection and intervention of the precancerous lesions and eradication of the Helicobacter pylori infection would also contributable [14].

Controversy over lymphadenectomy in GC surgery has persisted for several decades. Several large randomized studies and meta-analyses found that the D2 procedure was significantly associated with postoperative morbidity and mortality, rather than conferring a survival benefit [15–17]. Therefore, limited lymphadenectomy was utilized in a study of GC patients in Western countries [18]. However, not only the prognosis of surgery-only group but also surgery with chemotherapy group from Western trials was significantly poorer compared with that of surgery-only group from Eastern trials where more extensive lymphadenectomy (D2) has been a standard; those difference partly caused by the insufficient lymphadenectomy in Western trials [19–21]. Furthermore, the 15-year follow-up results of a Dutch trial indicate that D2 lymphadenectomy could decrease locoregional recurrence and GC-related death relative to D1 lymphadenectomy [22]. In our study, D2 lymphadenectomy was a positive prognostic factor in stage II and III patients, and D2 lymphadenectomy alone even cured some stage II patients. However, less than 50% of Chinese patients with stage II and III GC have undergone D2 lymphadenectomy, whereas more than 80% of Korean patients with the same stage underwent this surgery. This pattern explains why the survival of patients with stage II and III GC was significantly different between these two countries in initial analyses but was similar when the analyses were confined to the patients who underwent D2/D2+ lymphadenectomy.

In the past two decades, the Chinese anti-cancer association/Gastric Cancer Association have promoted a program of itinerant lectures on standard GC operation. Due to efforts to spread training for D2 lymphadenectomy in China, GC surgery has become more standardized in recent years. Our published data demonstrate that the proportion of D2/D2+ lymphadenectomy from 2000 to 2005 was approximately 10% but increased to nearly 40% after 2006, an expansion that was accompanied by an increase in the number of harvested lymph nodes and improved OS [7], demonstrating the survival benefit brought by D2 lymphadenectomy.

For safety reasons, Western guidelines recommend the performance of D2 surgery only in high-volume centres by experienced surgeons [23, 24]. Because the present results were from two large volume hospitals in two countries, there was no significant differences in morbidity and mortality even after D2 lymphadenectomy. However, it has been widely reported that D2 lymphadenectomy can be routinely performed with low morbidity and mortality in small-volume hospitals [17, 25, 26]. Therefore, there is no longer a sustainable argument against standard D2 gastrectomy in modern surgery for invasive GC, especially given the poor results of para-aortic lymphadenectomy from the JCOG 9501 trial [23, 27, 28]. Nonetheless, the training necessary for D2 gastrectomy and the quality of performance remain challenges need to be addressed.

Despite the receipt of D2 lymphadenectomy for resectable GC, about 40% of patients relapsed within three years of surgery [21, 27]. Therefore, various adjuvant treatment modalities have been investigated to reduce postoperative recurrence, with some Western clinical trials yielding favourable results [19, 20]. However, these results should be interpreted and applied cautiously to East Asian patients because lymphadenectomy in these studies was limited, and the results tended to vary by geographic region [29]. Two large randomized controlled trials in Asia (ACTS-GC trial and CLASSIC trial) have established the benefit role of adjuvant chemotherapy in stage II or III GC patients after D2 gastrectomy [21, 30].

In accordance with previous studies, our results demonstrate that chemotherapy can improve the prognosis of patients with stage III GC, even partly compensating for the absence of D2 lymphadenectomy in patients with stage II GC [19–21, 30]. In addition, adjuvant chemotherapy increased the OS of patients with distant metastasis. The proportion of stage III or IV patients receiving adjuvant therapy—those most likely to benefit from the addition of chemotherapy—was considerably smaller in China than in Korea. Several factors may account for this. We included patients from 2006 to 2010 in this study, but little evidence supporting the application of adjuvant therapy after D2 surgery occurred during that time. Therefore, some patients with curative surgery did not wish to receive chemotherapy because of the risk of toxic effects [29]. This is also true for Korean patients with stage II GC; low compliance of patients from both countries contributes to this phenomenon. Future attention should be paid in China to the spread of and education about chemotherapy, the invention of new drugs or regimens with lower toxicity, and the appropriate application of chemotherapy. With respect to lymphadenectomy and chemotherapy, factors controllable in clinical practice, only 20% of stage II-IV patients were given D2 lymphadenectomy and adjuvant chemotherapy although they account for nearly 80% of GC cases in China. In our study, both D2/D2+ and chemotherapy were selected as independent prognostic factors, and the best prognosis appeared in the patients treated by D2/D2+ with chemotherapy; if the proportion of patients with D2 lymphadenectomy and adjuvant chemotherapy increases, long-term survival will improve.

There are some limitations of this study. Firstly, selection bias, detection bias, and performance of analysis bias are possible in any retrospective study [31]. Secondly, our data are from only two institutions, one Chinese and one Korean; therefore, the data may not represent the general population well. However, the numbers of GC surgeries performed in these two institutions are large, and the patients’ origins cover large areas of China and Korea, which may serve as representative for two large populations. Thirdly, our study was confined to cases with gastrectomy, excluding patients who underwent non-resectional surgery.

In conclusion, many differences in tumour characteristics exist between these two countries. A high percentage of patients diagnosed GC in early stages and standardized treatment in Korea contributes to better survival than China. To improve survival outcomes in China, the promotion of an early screening program, training and spread of standard D2 lymphadenectomy, and appropriate applications of chemotherapy are necessary.

## MATERIALS AND METHODS

### Patients

**Figure 5:**
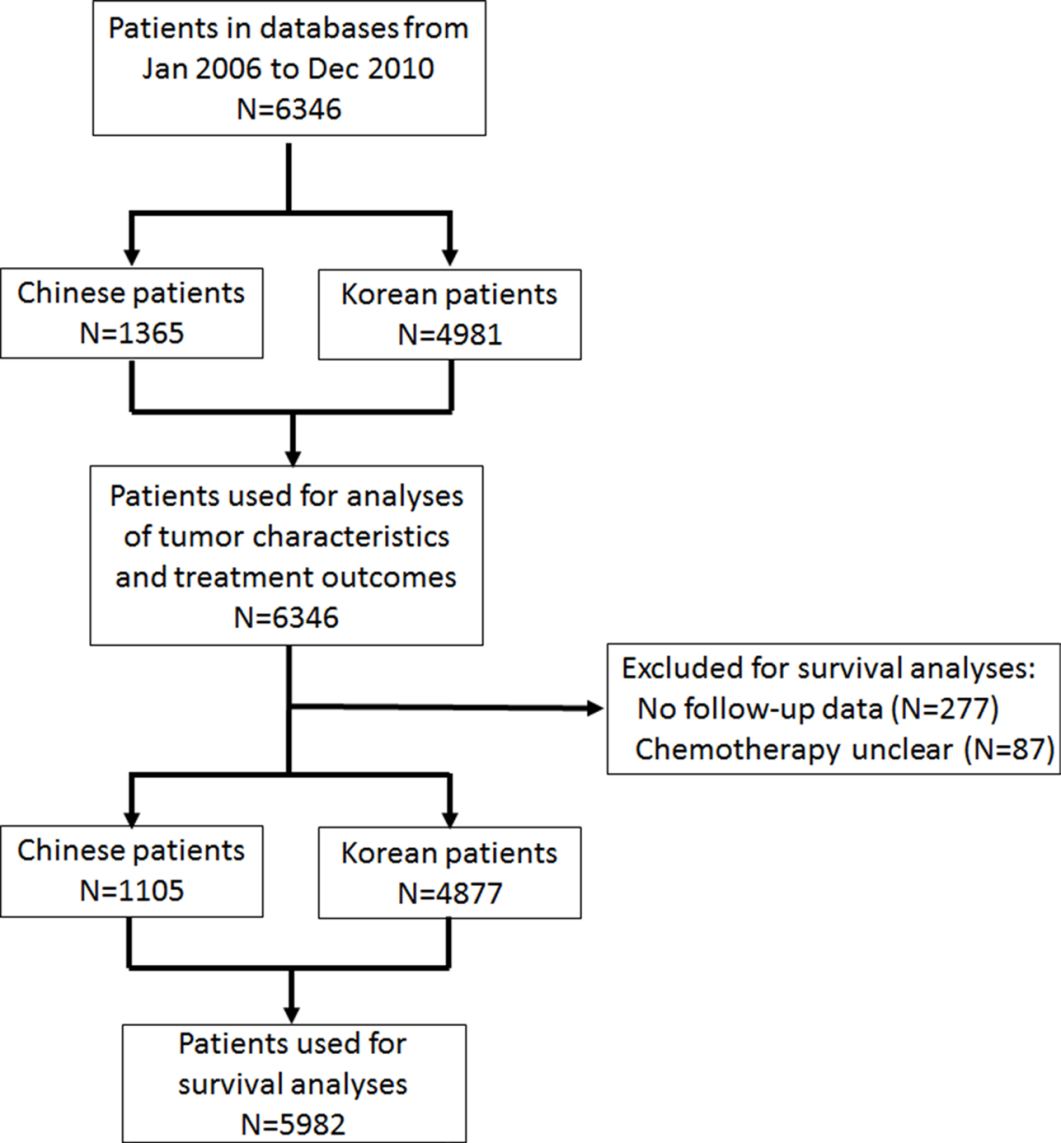
Flow chart showing selection procedure of patients

This study consisted of 6346 GC patients, 1365 Chinese and 4981 Korean, diagnosed between January 2006 and December 2010. Data were extracted from the databases of West China Hospital, Sichuan University, and Severance Hospital, Yonsei University Health System, and analysed retrospectively (Figure 5). The diagnosis of gastric adenocarcinoma for all patients was confirmed by upper endoscopy and biopsy. Inclusion criteria were the following: all cases of early and advanced GC, both curative and palliative gastrectomies, and patients with total or subtotal gastrectomies. Non-resectional surgeries, however, such as bypass surgery, gastrostomy, or jejunostomy, were excluded. Patients with other gastric pathologies, such as lymphoma, gastrointestinal stromal tumour or adenosquamous carcinoma, previous malignancies, remnant GC, or those treated by wedge resection or endoscopic resection were also excluded. The West China Hospital Research Ethics Committee and Institutional Review Board of Severance Hospital, Yonsei University Health System, approved retrospective analyses of anonymous data (4-2015-0647). Signed patient informed consent was waived because of the retrospective nature of the analysis.

### Treatments

All patients underwent gastrectomy with D1, D1+, D2, or D2+ lymphadenectomy for GC as defined by the Japanese Classification of Gastric Carcinoma [32]. Total or subtotal gastrectomy was performed according to the location of the primary lesion. Billroth I, Billroth II, or Roux-en-Y anastomosis with hand-sewn or mechanical staples was performed to reconstruct the digestive tract after distal gastrectomy. Esophagogastric anastomosis was used after proximal gastrectomy, and Roux-en-Y esophagojejunostomy was utilized for total gastrectomy. Chemotherapy treatments consisted of fluoropyrimidine alone or a fluoropyrimidine/platinum-based regimen.

### Outcomes measurements

Clinicopathologic features, treatment outcomes, morbidity and mortality (according to the Clavien-Dindo Classification), and overall survival (OS) were compared [33]. Patients underwent follow-ups conducted by telephone calls, letters, or outpatient visits. Survival status at the last follow-up for Korean patients was also based on data registered with the Korean National Cancer Center. The follow-up information was updated in December 2014 for Chinese patients and March 2014 for Korean patients. The overall follow-up rate was 95.6% (6069/6346). OS was calculated from the date of operation until the date of death or the last follow-up. The mean follow-up duration was 69.3 ± 20.3 months in Chinese patients and 56.2 ± 16.9 in Korean patients. All terminologies were based on the Japanese Classification of Gastric Carcinoma [34].

### Statistical analysis

The assumption of normality for continuous variables was assessed by the Kolmogorov-Smirnov test. All data are reported as mean ± standard deviation for continuous variable or frequency (percentage) for categorical variables. An independent two-sample *t*-test, Pearson's chi-square test (or Fisher's exact test), or Spearman's test were used to compare differences between the two countries, as appropriate. Survival rates were calculated by Kaplan-Meier estimator and compared by the log-rank test. Univariable or multivariable Cox proportional hazards regression models were used to identify independent prognostic factors. Variables included in multivariable models were selected by the forward method with likelihood ratio (two-tailed *p* ≤ 0.05). Two-tailed *p*-values of less than 0.05 were considered statistically significant. Statistical analyses were performed by using SPSS 19.0 software (SPSS, Chicago, IL, USA).
